# Declining Trends in Gastrointestinal Parasitic Infections Among Nepalese School Children: A Systematic Review and Meta‐Analysis (2004–2022)

**DOI:** 10.1002/puh2.70180

**Published:** 2025-12-28

**Authors:** Jitendra Gautam, Niten Bharati, Shristi Bhandari, Darwin Niroula, Anisha K. C., Pitambar Dhakal, Kishor Pandey, Oskar Nowak, Rajendra Prasad Parajuli

**Affiliations:** ^1^ Central Department of Zoology Institute of Science and Technology Tribhuvan University, Kathmandu Kirtipur Nepal; ^2^ Adam Mickiewicz University in Poznań Poznań Poland; ^3^ Herbert Wertheim School of Public Health and Human Longevity Science University of California San Diego (UCSD) San Diego California USA

**Keywords:** gastrointestinal parasites | meta‐analysis | Nepal | prevalence | school‐going children | temporal trends

## Abstract

**Background:**

Gastrointestinal parasitic infections (GIPs) remain a major health concern in low‐income countries, particularly among school‐aged children. Nepal's national deworming program, launched in 2004, has significantly improved in controlling GIPs, yet they persist in many areas. This review synthesizes evidence on the prevalence and temporal trends of GIPs among schoolchildren in Nepal.

**Methods:**

PubMed, Google Scholar, and regional databases were searched for studies published between 2004 and 2022, reporting the prevalence of GIPs among children aged 5–17 years. Studies providing information on sample size, location, and clear diagnostic methods were considered eligible for analysis. Pooled prevalence estimates were calculated using a random‐effects model (DerSimonian–Laird) with logit transformation. Heterogeneity (*I* ^2^, *τ* ^2^) and temporal trends (meta‐regression) were assessed.

**Results:**

Twenty‐five studies comprising 17,628 stool samples were included, of which 7091 (40.2%) tested positive for GIPs. The pooled prevalence was 28.6% (95% CI: 21.7%–36.7%), with substantial heterogeneity. Prevalence declined significantly over time (OR = 0.58, 95% CI: 0.41–0.81), from 43.4% (2004–2010) to 24.2% (2010–2016), followed by a slight increase to 28.4% (2016–2022). Prevalence was similar by sex, with approximately 12.7%, and urban areas had a slightly higher prevalence (29.0%) than did rural areas (27.9%). Helminth infections (20.0%) were more common than protozoans (9.9%), and in 7.7% (95% CI: 5.6%–9.7%) of the children, polyparasitism was recorded.

**Conclusion:**

GIPs among Nepalese schoolchildren have declined notably over the past two decades but remain a persistent concern. Continued disparities highlight the need for sustained deworming, sanitation, and region‐specific monitoring.

## Graphical abstract

This meta‐analysis of 25 studies (17,628 children) shows a substantial decline in gastrointestinal parasitic infections in Nepal from 43.4% to 24.2% between 2004 and 2022. Helminths (20.0%) remained more common than protozoa (9.9%), with slightly higher prevalence in urban (29.0%) than rural areas (27.9%).

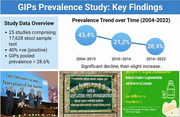

## 1 Introduction

Soil‐transmitted helminth (STH) infection remains one of the most prevalent neglected tropical diseases, affecting an estimated 1.5 billion people across Africa, the Americas, and Asia [1, 2, 3]. Approximately one‐fourth (24%) of the global population in 101 countries is infected with these parasites. Among them, *Ascaris lumbricoides* (ascariasis) is the most common, affecting approximately 819 million individuals, whereas *Trichuris trichiura* infects approximately 464 million individuals, together contributing to an estimated 0.64 million disability‐adjusted life years (DALYs) lost annually [4, 5, 6]. Parasitic infections are particularly widespread in developing countries, where various geographic, environmental, and socioeconomic factors promote transmission. In contrast, intestinal helminths are less prevalent in industrialized nations, where protozoan infections are comparatively more common [7, 8, 9]. Among protozoans, *Entamoeba* species are the third most common cause of intestinal infection globally, with an estimated prevalence of approximately 3.5% [10, 11]. Across developing regions, gastrointestinal parasitic infections (GIPs) caused by helminths and protozoa remain among the most common communicable diseases, imposing significant health burdens [12]. In South Asia, including Nepal, GIPs are especially prevalent among children across diverse ethnic groups, adversely affecting their nutrition, growth, and cognitive development [9, 13, 14].

GIPs have remained a persistent public health concern in Nepal over the past two decades, particularly among school‐aged children [15, 16]. These infections contribute substantially to morbidity and mortality and impair children's physical growth and cognitive development [2, 3, 17]. Earlier studies from the 1960s to the 1990s reported high infection rates in both rural and urban settings, largely attributable to poor sanitation and hygiene practices [10, 18, 19]. Since then, Nepal has made notable progress in sanitation, hygiene, education, and socioeconomic development [20]. By 2019, all 77 districts had been declared open defecation free (ODF), and household toilet coverage had increased from 22% in 1995/96 to over 56% [3, 21, 22]. Over the past three decades, national programs and campaigns, such as mass deworming, vitamin A supplementation, and the provision of afternoon breakfast to children at school, have further supported improvements in children's health and nutrition [3, 17, 23]. Since the launch of Nepal's national deworming program in 2004, the overall burden of GIPs has shown a declining trend [24]. However, despite mass deworming (i.e., >95% coverage) in tandem with vitamin A supplementation for preschool children, the reported prevalence still varies widely—from approximately 10.9% to over 40%—depending on the geographic area and population studied [25, 26]. Robust, long‐term surveillance data for evaluating national trends remain limited. Previous systematic reviews, including Kunwar et al. [15], examined GIPs among Nepalese school children up to 2015 and reported an overall decline, with a higher prevalence persisting in specific ethnic and geographic pockets [15]. Since then, several major policy and infrastructural reforms have been implemented, including the expansion of deworming programs, nationwide sanitation and hygiene campaigns, and strengthened school health and education initiatives [11, 27]. These developments likely contributed to further reductions in GIPs, underscoring the need for an updated synthesis incorporating nearly a decade of additional evidence.

Evaluating the sustained impact of these interventions requires assessing whether infection rates have continued to decline consistently [17]. Furthermore, rapid urbanization, population mobility, climate variability, and advances in water and sanitation infrastructure may have reshaped the epidemiology of GIPs [28, 29, 30]. This review therefore aims to capture these evolving patterns up to 2022, providing an updated and comprehensive understanding of infection prevalence in Nepal. Recent studies employing advanced diagnostic methods may also refine pooled prevalence estimates and reveal new insights into polyparasitism patterns. To address these gaps, we conducted a systematic review and meta‐analysis to examine temporal trends in GIPs among school‐aged children in Nepal from 2004 to 2022. Our analysis explored variations by sex, parasite type, area of residence, and polyparasitism, offering evidence to guide more targeted and effective prevention and control strategies.

## 2 Materials and Methods

This review was conducted in accordance with the Preferred Reporting Items for Systematic Reviews and Meta‐Analyses (PRISMA) guidelines and adheres to established standards for performing systematic reviews and meta‐analyses.

### 2.1 Geographical Context of Nepal

**FIGURE 1:**
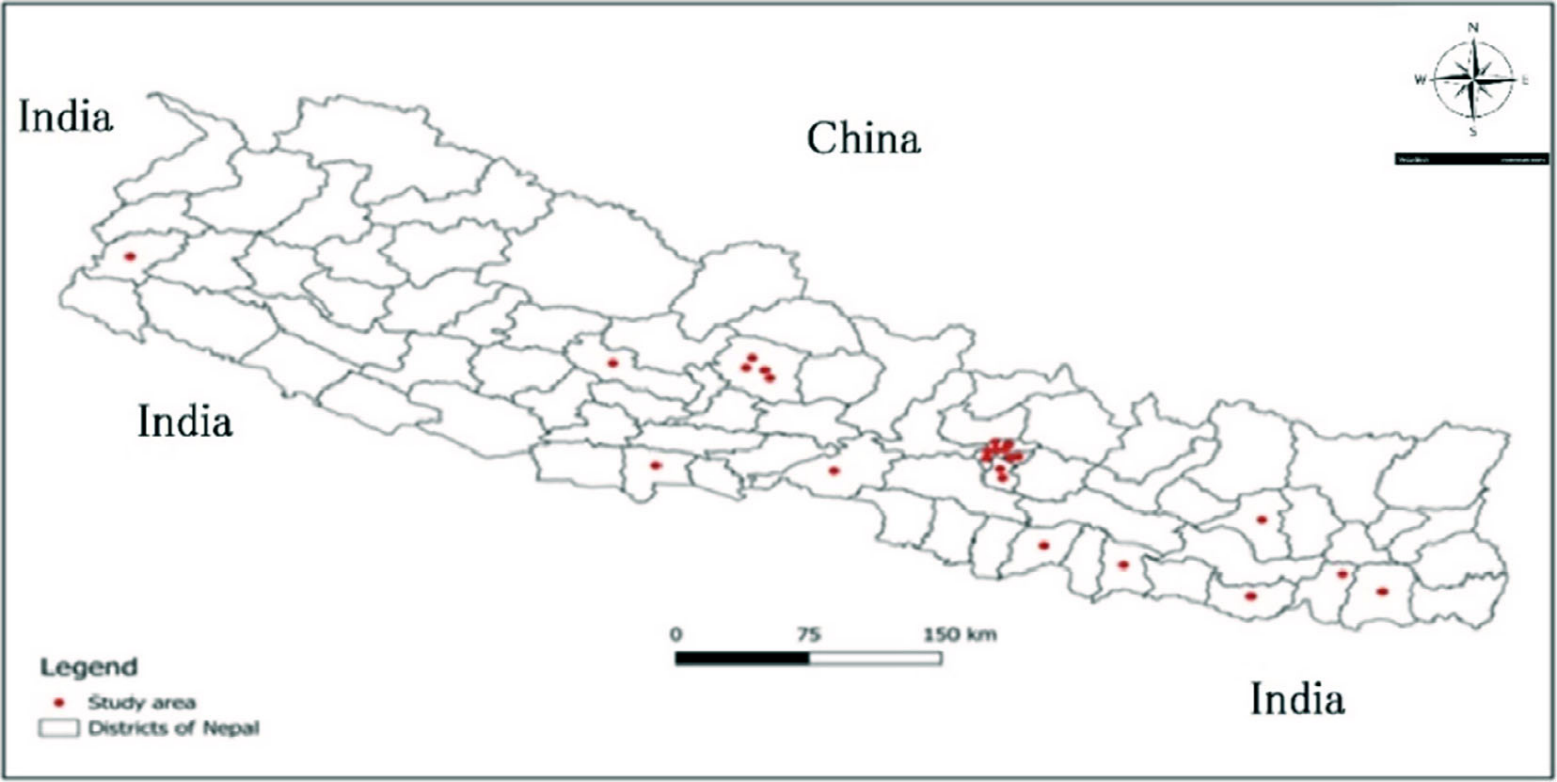
Overview of the study area, with dots representing sampling locations within Nepal.

Nepal, located in South Asia, is classified as one of the world's least developed countries. It is bordered by India to the east, west, and south and by China to the north [31] (Figure 1). The country experiences heavy monsoon rains, with an average annual precipitation of approximately 1400 mm. Geographically, Nepal is divided into three ecological zones: the Himalayan highlands, the midhill region, and the Terai lowlands [17]. These zones vary considerably in altitude—from 8848 m at the highest point to 59 m at the lowest point—and exhibit distinct differences in climate, vegetation, and snowfall [3].

As of 2024, Nepal's gross domestic product (GDP) was estimated to be approximately $43.42 billion, with a per capita income of $1389.43 [32]. The total population is 30 million, and approximately 20% of the population lives below the poverty line [33]. Approximately 6.37 million students are enrolled across Nepal's primary, lower secondary, secondary, and higher secondary levels [34, 35], representing roughly one‐quarter of the national population. In addition, about 24% of Nepal's population consists of adolescents aged 10–19 years [36], covering much of the school‐age group. In addition to school‐based delivery, Nepal's national deworming program is also implemented through biannual community campaigns integrated with the national vitamin A program, whereby female community health volunteers and local health facilities distribute albendazole to all eligible children, including those who are out of school or have dropped out. These diverse ecological and socioeconomic conditions influence sanitation, hygiene, and health infrastructure across Nepal, shaping GIP transmission patterns and guiding targeted interventions.

### 2.2 Eligibility Criteria

This systematic review included all available surveys conducted in Nepal from 2004 to 2022 that reported the overall GIPs infections among school‐aged children, primarily those aged 5–17 years and enrolled in grades 1–10.

### 2.3 Information Sources and Literature Search

**FIGURE 2:**
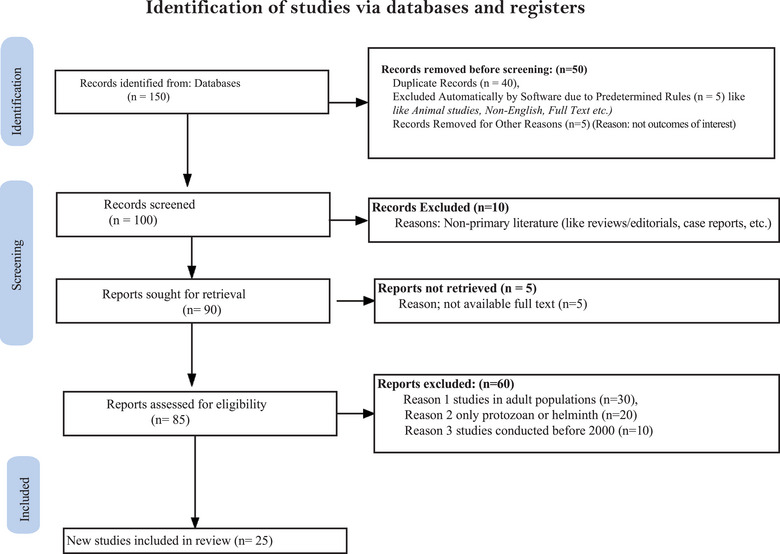
Flow diagram showing the literature selection process.

We conducted a comprehensive search of studies published or reported between 2004 and 2022 via PubMed and Google Scholar. The Boolean search strings used were as follows: (“parasite” OR “parasitic infection” OR “helminth” OR “protozoa” OR “soil‐transmitted helminth” OR “intestinal parasite”) AND (“school children” OR “student” OR “child” OR “childhood”) AND (“Nepal”). After the initial screening, we also manually reviewed the reference lists of all relevant articles to identify additional sources. To capture studies published in regional peer‐reviewed journals that may not be indexed online, one author (N.B.) searched physical collections in local libraries (where four relevant MSc theses were located but later excluded due to missing demographic details). A detailed description of the search strategy is provided in Figure 2.

### 2.4 Study Selection

The study selection process is illustrated in Figure 2. To be eligible for inclusion in this systematic review, studies had to report key methodological and participant details, including children's age and sex, study location, sample size, season or timing of data collection, date of stool sample collection, and the laboratory techniques used for parasitological examination. Studies were excluded if they involved adult participants, were conducted before 2000, focused exclusively on either helminth or protozoan infections with fewer than 100 samples, or included only children seeking care for gastrointestinal symptoms in clinical or hospital settings.

### 2.5 Data Collection Process

Data were independently extracted using standardized data collection forms by the two co‐authors (J.G. and N.B.), and any discrepancies were resolved through discussion and consensus. Key information included the number of children confirmed to have GIPs through laboratory testing; the total number of children who submitted stool samples; and details such as sex, age, year and location of sample collection, diagnostic methods used, season of data collection (classified as rainy or non‐rainy), and area of residence (urban or rural). As a single stool sample could contain multiple helminth and/or protozoan species, the total number of each parasite type identified per survey was also recorded. The classification of the study location as “rural” was based on the authors’ explicit designation; if unspecified, areas described as villages situated a significant distance from district headquarters were also considered rural. June–September, corresponding to Nepal's peak rainfall months, was defined as the “rainy season,” whereas the remaining months were categorized as “non‐rainy.” The detection of more than one intestinal parasite species—helminth or protozoan—in a single host was classified as polyparasitism.

### 2.6 Risk of Bias in Individual Studies and Across Studies

A formal quality assessment and risk of bias analysis were not performed for individual studies, as the majority were basic prevalence surveys reporting the proportion of infected children by demographic characteristics. Nonetheless, as outlined in the study selection criteria, all included studies were required to meet a predefined set of eligibility standards. In randomized controlled trials, publication or reporting bias is often evaluated using funnel plots and statistical tests to determine whether smaller studies with null or negative outcomes are underreported. However, the impact of publication bias on prevalence studies, particularly those reporting extreme results, has not been sufficiently addressed in the literature. Although we did not apply traditional statistical or visual methods for detecting publication bias in this meta‐analysis, we included a scatter plot displaying the log odds of GIP against the study sample size. This visualization offers insight into potential patterns related to study size and publication tendencies.

### 2.7 Statistical Analyses

The meta‐analysis was conducted via the metaprop function of the meta package in R statistical software. For each included study, the prevalence (*π*) was determined by dividing the number of positive cases by the total number of individuals sampled. The standard error (SE) of the prevalence was calculated using the following formula:

$$\mathrm{SE}=\sqrt{\frac{\pi \;\left(1-\pi \right)}{n}}$$

where *n* denotes the total number of samples in each study. To address variability across studies, a random‐effect model was employed using the restricted maximum likelihood (REML) method. Proportion data were logit‐transformed (PLO) to stabilize variance and improve the reliability of pooled estimates. A meta‐regression analysis was performed to examine temporal trends, where earlier study periods served as reference categories; an odds ratio (OR) < 1 indicated a decline in infection prevalence over time.

### 2.8 Heterogeneity Assessment

To assess variability across studies, heterogeneity was measured using the *I* ^2^ statistic, which represents the percentage of total variation attributable to differences between studies rather than chance. The tau‐squared *(τ* ^2^) value was also computed to estimate the variance among the true effect sizes. Additionally, Cochran's *Q* test was applied to determine whether the observed heterogeneity was statistically significant. To address the high heterogeneity, subgroup analyses by study period, sex, residence, and parasite type, along with sensitivity analyses, were conducted to explore potential sources of variation.

### 2.9 Visualization of Trends

Bubble plots were used to visualize the associations between the prevalence estimates and the year of study, with the size of each bubble corresponding to the study's sample size. Trend lines representing meta‐regression estimates and their 95% confidence intervals (CIs) were generated by applying the inverse logit transformation to the predicted values. Separate trend analyses were performed for overall prevalence, as well as for the male and female subgroups.

### 2.10 Summary Measures and Visualization

The pooled prevalence, along with its 95% CI, was calculated using the inverse variance approach. Additionally, a prediction interval was estimated to indicate the likely range of the predicted prevalence. A forest plot was created to display the prevalence estimates from individual studies, the overall pooled estimate, and measures of heterogeneity.

### 2.11 Software and Statistical Tools

All statistical analyses were conducted using the meta package in R software (version 2024.12.0+467), a freely available, open‐source statistical environment developed by the R Foundation for Statistical Computing (Vienna, Austria; https://www.r‐project.org/). Confidence intervals for proportions reported in individual studies were calculated via the Clopper–Pearson exact method. To explore trends, we performed meta‐regression of prevalence estimates via generalized linear models weighted by sample size. Owing to the limited number of studies per individual year, studies were grouped into broader time intervals for analysis. The prevalence proportions (*p*), representing the fraction of participants with GIPs, were transformed using the logit function (log odds). A linear regression was then applied to these logit‐transformed values over time, modeling the relationship between prevalence and the time variable *t*.

## 3 Results

### 3.1 Features of the Study

A total of 25 surveys met the eligibility criteria (Figure 2). The geographic distribution of the study sites included in the analysis is presented in the accompanying map. Nineteen of the studies reported the use of the formal ether concentration technique for sample processing, whereas six relied exclusively on the direct wet mount method. Altogether, these studies examined 17,628 stool samples, with all participants aged between 5 and 17 years as part of school‐based prevalence surveys. A wide range of gastrointestinal parasites were identified across the included studies. Helminths detected included *A. lumbricoides*, hookworm, *T. trichiura*, *Strongyloides stercoralis*, *Hymenolepis nana*, *Hymenolepis diminuta*, *Taenia* spp., *Enterobius vermicularis*, and *Vampirolepis nana*. Protozoan parasites reported were *Entamoeba histolytica*, *Giardia lamblia, Balantidium hominis*, *Entamoeba hartmanni, Endolimax nana, Cyclospora* spp., *Blastocystis hominis, Iodamoeba bütschlii, Entamoeba coli*, and *Amiroles nana*. Several studies also documented polyparasitism, where children harbored multiple helminths and/or protozoan species in a single stool sample.

**FIGURE 3:**
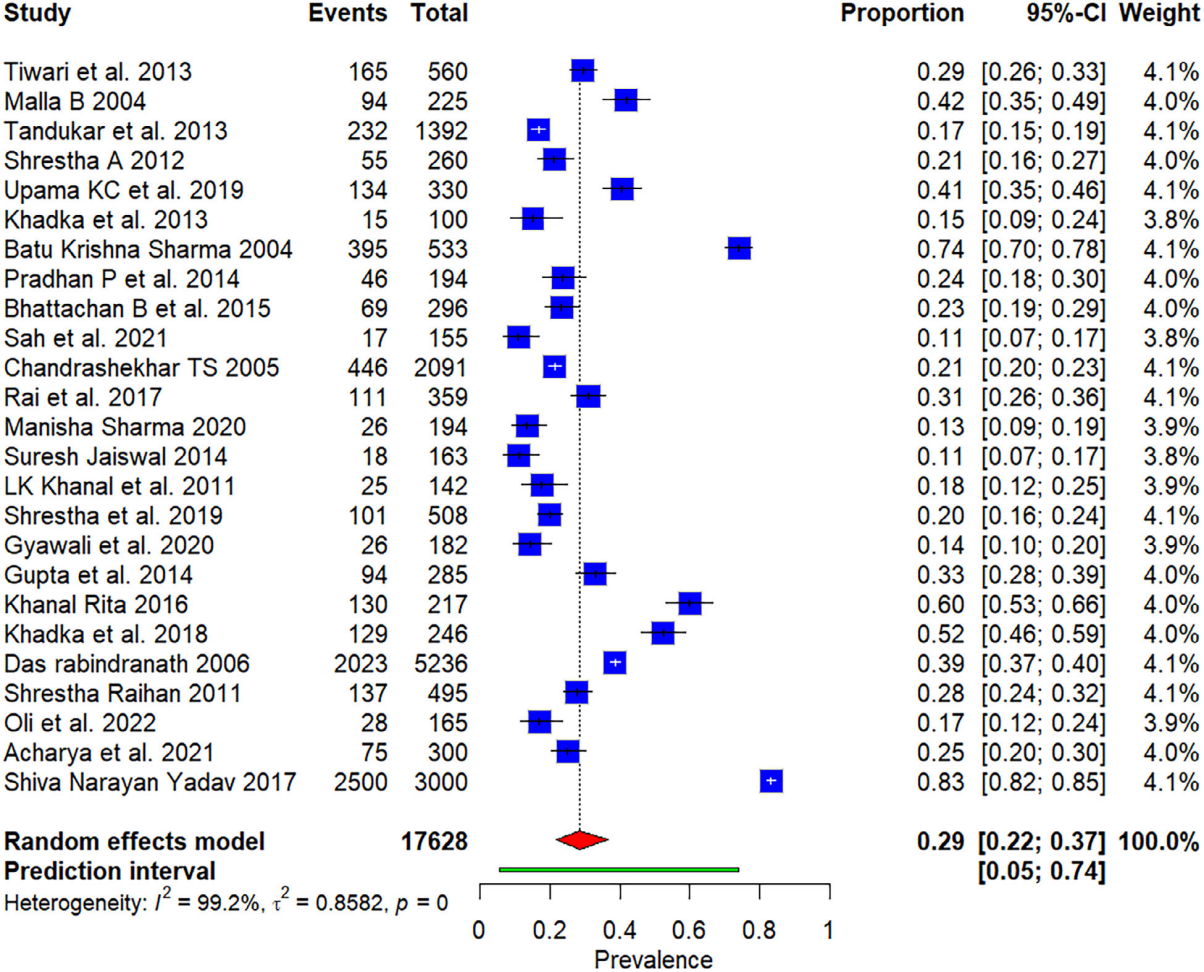
Forest plot showing the pooled prevalence of gastrointestinal parasitic infections.

The meta‐analysis included 25 studies, encompassing a total of 17,628 participants, among whom 7091 cases (40.2%) tested positive for GIPs. Using a random effects model, the pooled prevalence was calculated to be 28.64% (95% CI: 21.74%–36.71%), indicating a significant burden of GIPs within Nepal. The prediction interval ranged from 5.40% to 73.85%, indicating a wide range of prevalence estimates. A very high level of heterogeneity was identified (*I* ^2^ = 99.2%), which was statistically significant (*Q* = 3052.32, *p* < 0.001), reflecting notable differences across the included studies (Figure 3). The *τ* ^2^ statistic, estimated at 0.8582, also indicated substantial variability between studies.

In the plot, the blue squares represent individual study results, the red diamonds denote the pooled estimate, and the green shading indicates the prediction interval.

#### 3.1.1 Trends in the Overall Prevalence of GIPs From 2004–2022

**FIGURE 4:**
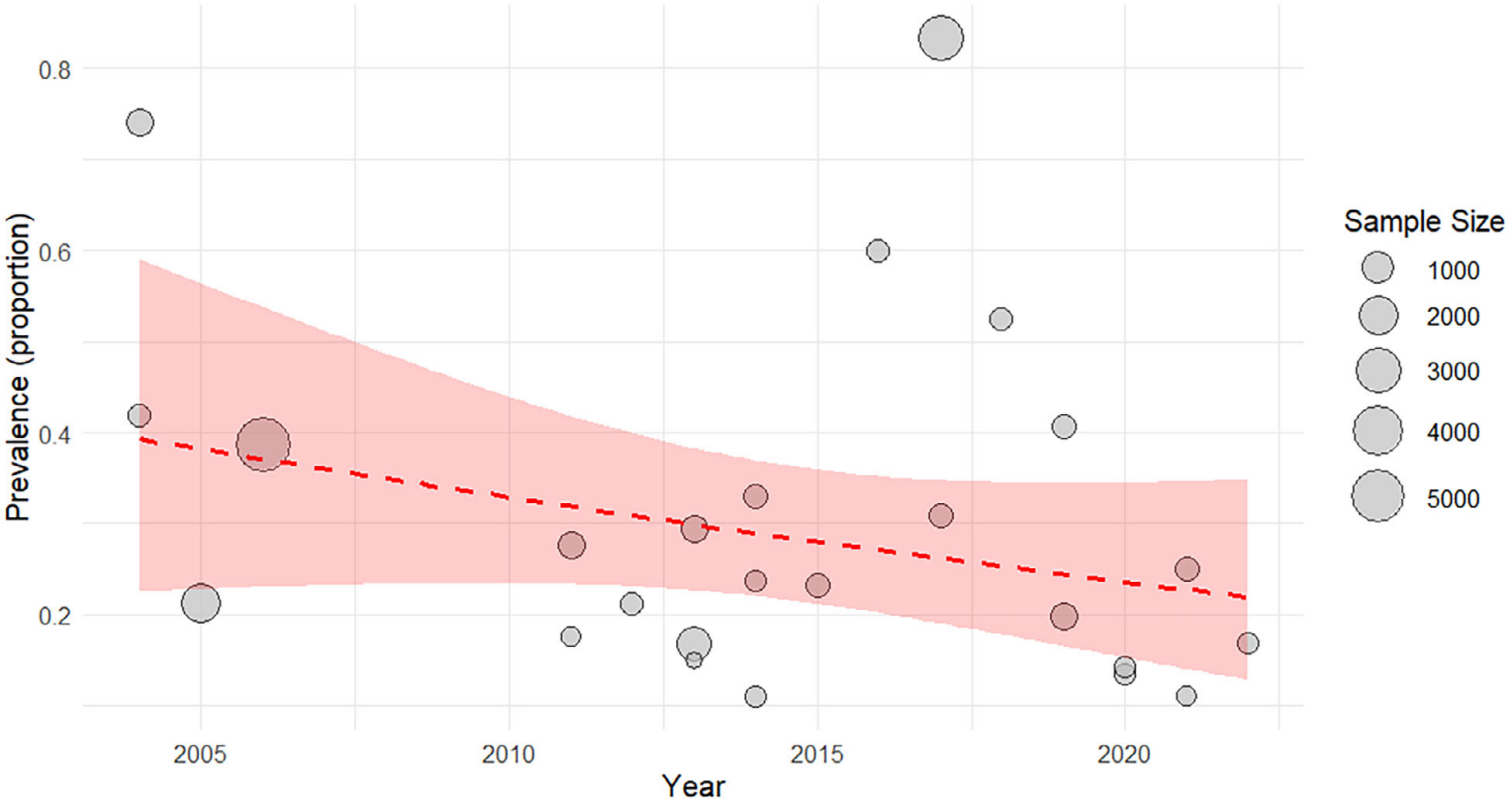
Overall trend in the prevalence of parasites over time.

Between 2004 and 2010, the pooled prevalence of GIPs was 43.4% (95% CI: 26.4%–62.2%). This percentage declined markedly to 24.2 (95% CI: 18.2%–31.3%) during the 2010–2016 period, indicating a significant decrease in infection burden (Figure 4). However, a modest increase was noted from 2016 to 2022, with the prevalence reaching 28.4% (95% CI: 12.9%–51.6%). The wide confidence interval in the latter period points to considerable heterogeneity, which may be linked to differences in study populations or the impact of large‐scale studies included in that timeframe.

#### 3.1.2 Prevalence of Parasitic Infections Stratified by Sex Across Time Periods

**FIGURE 5:**
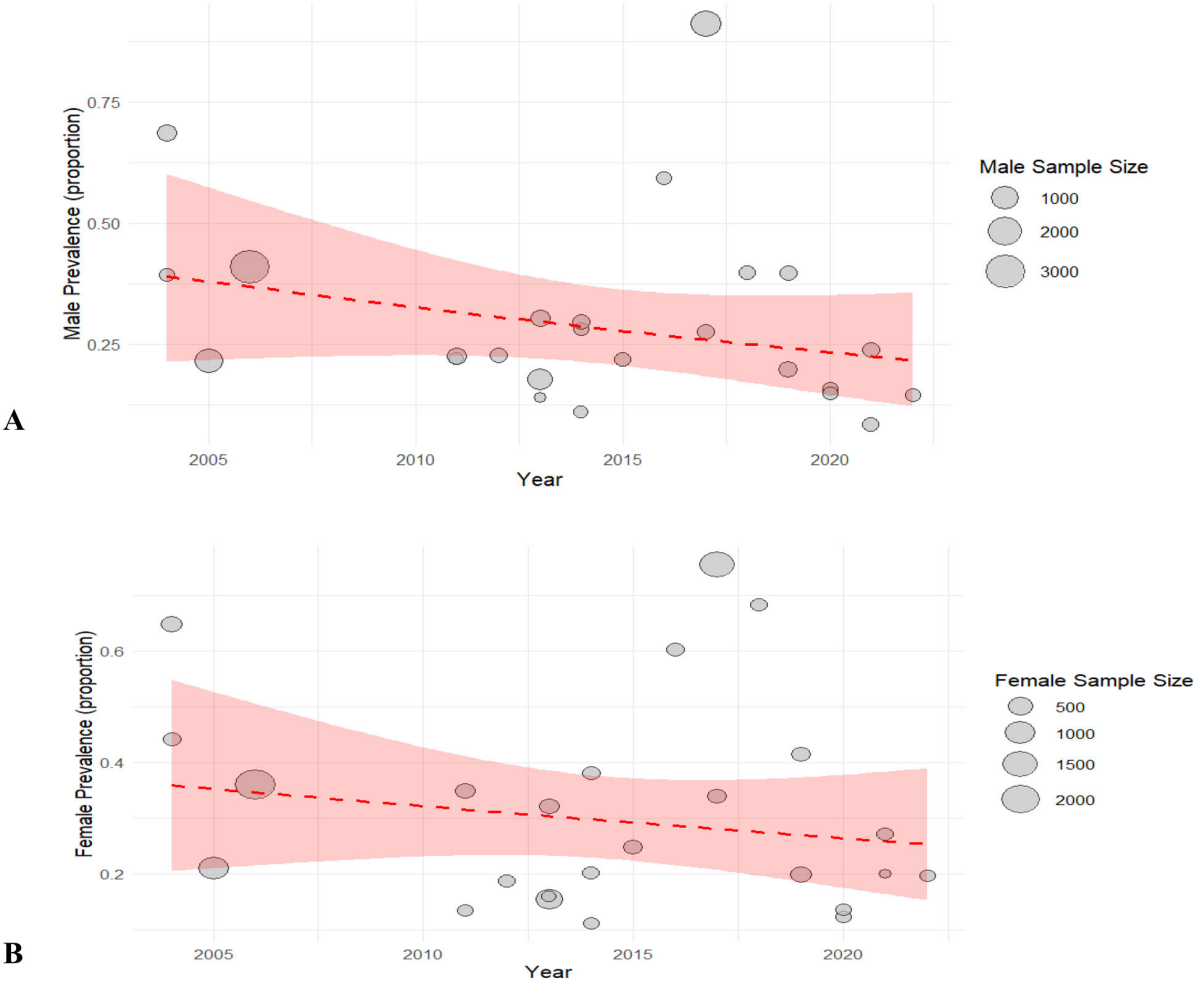
Overall prevalence trend of gastrointestinal parasites over the years (2004–2022) by sex (A: male: B: female).

A sex‐ and time‐stratified meta‐analysis of GIP incidence in Nepal from 2004 to 2022 indicated a general downward trend, with similar patterns observed earlier in overall prevalence. These groupings from 2004 to 2010, 2010 to 2016, and 2016 to 2022 were selected to reflect major public health milestones: the initiation of national deworming programs (2004–2010), rapid expansion of sanitation and ODF initiatives (2010–2016), and the period of intensified surveillance and increased publication of parasitological studies (2016–2022). Among males, the pooled prevalence was 41.8% (95% CI: 26.0%–59.5%) from 2004 to 2010, declined to 24.7% (95% CI: 19.2%–31.1%) from 2010 to 2016, and rose slightly to 27.9% (95% CI: 10.9%–55.0%) from 2016 to 2022 (Figure 5A). For females, the prevalence rates were 40.4% (95% CI: 25.7%–57.1%) from 2004 to 2010, 24.6% (95% CI: 17.4%–33.6%) from 2010 to 2016, and 31.1% (95% CI: 16.5%–50.9%) from 2016 to 2022 (Figure 5B). On the basis of an assumed baseline prevalence of 20% and an OR of 0.58 (95% CI: 0.41%–0.81%) for both sexes, the adjusted prevalence estimate was 12.7% (95% CI: 9.3%–16.8%) for both males and females. The broad confidence intervals, especially in the most recent time period, point to significant heterogeneity likely influenced by large or methodologically diverse studies.

#### 3.1.3 Trends in Protozoan and Helminth Parasite Infections in Nepal (2004–2022)

**FIGURE 6:**
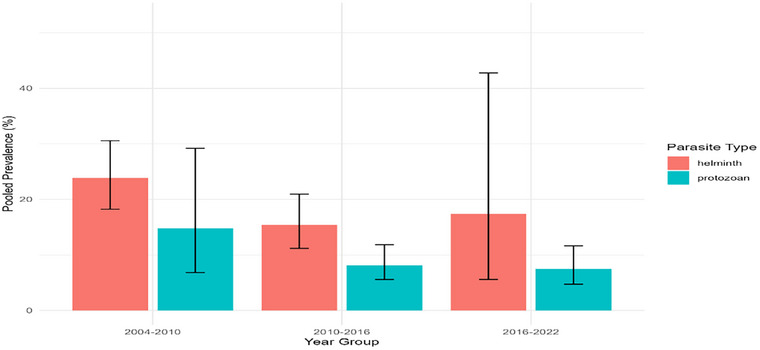
Overall trend in the prevalence of parasites across the three study periods (2004–2022).

An analysis of 25 studies conducted in Nepal between 2004 and 2022 highlighted contrasting temporal patterns in protozoan and helminth infections (HIs) among children. Although the pooled prevalence of both parasite types declined over time, protozoan infections showed a sharper reduction—from 14.8% (95% CI: 6.8%–29.2%) from 2004 to 2010 to 7.4% (95% CI: 4.7%–11.6%) from 2016 to 2022. The helminth prevalence also declined, albeit less markedly, from 23.8% (95% CI: 18.2%–30.6%) to 17.4% (95% CI: 5.6%–42.8%) over the same period (Figure 6).

Logistic regression analysis revealed a significant downward trend for protozoan infections (*β* = −0.077, *p* < 0.001), whereas HIs showed an unexpected upward trend (*β* = 0.074, *p* < 0.001). A significant interaction term (*p* < 0.001) confirmed the divergent trajectories between these two parasite groups. Across all timeframes, HIs remained more prevalent than protozoan infections did, with average prevalence estimates of 20.0% and 9.9%, respectively. Considerable heterogeneity—especially during the 2016–2022 period—suggests that differences in the regional context or study scale may have contributed to the observed variability.

#### 3.1.4 Trends in the Prevalence of GIPs by Area of Residence (Urban vs. Rural)

**FIGURE 7:**
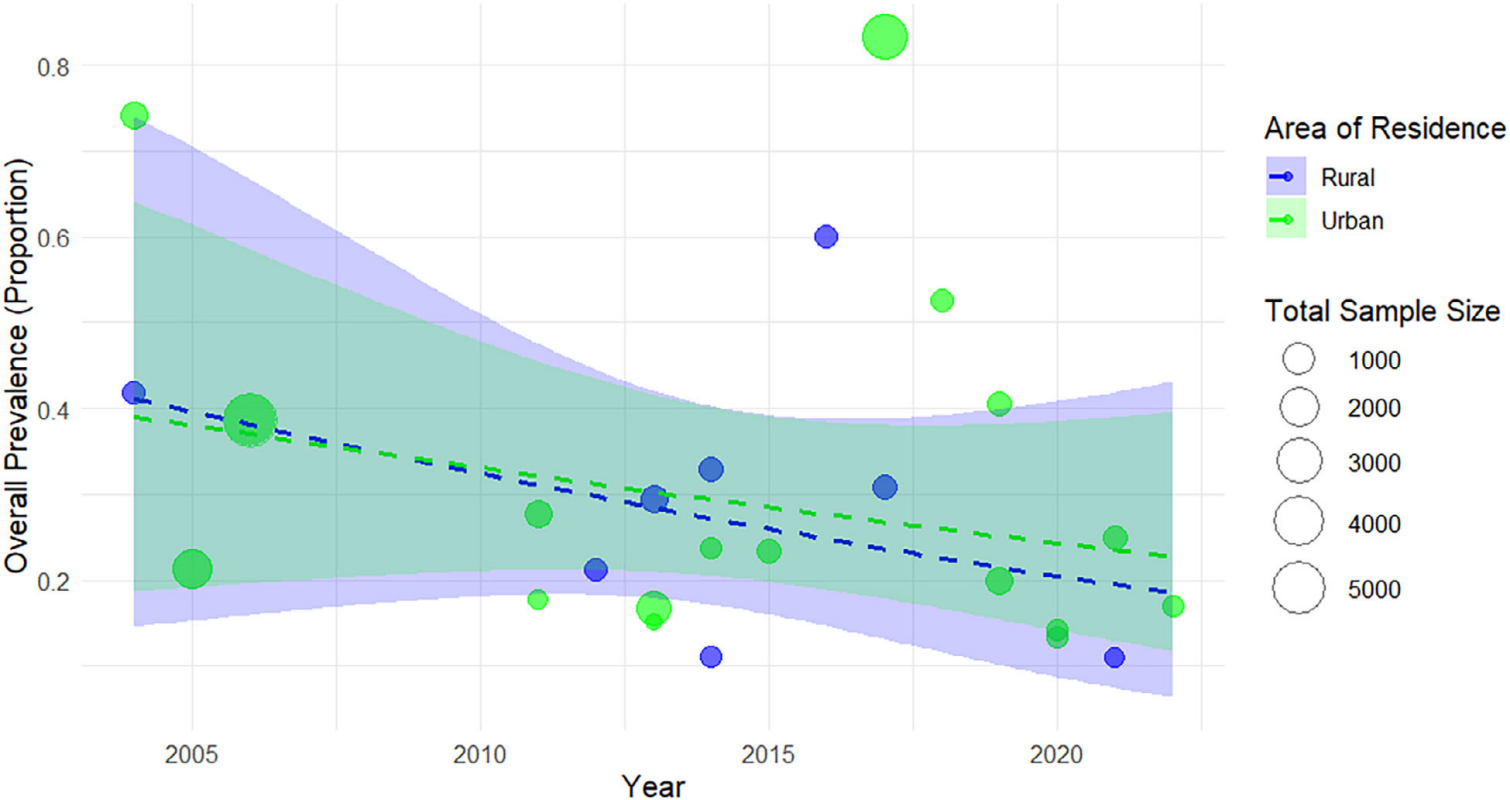
Overall trend in the prevalence of parasites over time (2004–2022) by area of residence.

In urban settings, the combined prevalence of GIPs was estimated to be 29.0% (95% CI: 19.2%–41.4%), which was marginally higher than the 27.9% (95% CI: 19.8%–37.8%) reported for rural areas (Figure 7). The broad confidence intervals in both groups reflect substantial heterogeneity among the included studies, likely influenced by differences in study design, sample size, and regional disparities in sanitation conditions and access to healthcare services.

## 4 Discussion

This meta‐analysis and systematic review offer a thorough evaluation of the downward trends in GIPs among Nepalese school children between 2004 and 2022. Through the combination of data from 25 studies with a total of 17,628 participants, the study provides important information about the demographic, parasitological, and temporal trends of GIPs in Nepal. Despite considerable variation throughout studies, the aggregate findings show a significant decrease in GIP prevalence over the last 18 years, which is indicative of the likely benefits of deworming, health education, and better sanitation. The combined prevalence of GIPs was 28.64% (95% CI: 21.74%–36.71%), indicating that GIPs still affect almost one in three Nepalese school children. Despite the significant total burden, there was a discernible downward trend, from 43.4% (2004–2010) to 24.2% (2010–2016) before a slight increase to 28.4% (2016–2022). This decrease is in accordance with past regional and national reports that showed how well deworming campaigns, better hygiene promotion, and school‐based health initiatives worked in Nepal [16, 17, 37]. Notably, the slight rise in helminth prevalence observed during 2016–2022 corresponds with a period of expanding parasitological research, improved diagnostic sensitivity, and an increased focus on high‐burden or underserved populations. Several surveys conducted during this period adopted concentration‐based diagnostic methods capable of detecting low‐intensity infections that earlier direct‐smear approaches may have missed, thereby increasing detectable prevalence. Although Nepal's deworming program continued without interruption, temporary operational challenges during the COVID‐19 pandemic, along with regional differences in sanitation and water access, may have contributed to localized increases. These methodological and contextual factors together provide a more plausible explanation for the modest elevation in prevalence than changes in program implementation. In a similar vein, Kunwar et al. noted a significant decline in GIPs in school‐age children from 2000 to 2015 [24]. However, increasing trends have been noted in other nations, such as Egypt [2, 38], Turkey [39], Ethiopia [12, 40], and Tanzania and Vietnam [41], among school children, mostly owing to poor water quality and inadequate sanitation and no provision of continued deworming initiatives in schools [5, 12, 40, 42, 43, 44, 45].

In contrast to the anticipated decreasing trend, there was a minor increase in prevalence between 2016 and 2022. Persistent infection rates above 30% have been reported in some community‐based surveys from Terai and isolated hill locations in Nepal [13, 20, 46, 47]. These findings may reflect poor water quality and insufficient sanitation. Regional differences in hygienic infrastructure, population migration, and the effects of climate on parasite transmission could all be contributing factors to recent revival. The substantial heterogeneity (*I* ^2^ = 99.2%) and broad prediction interval (5.40%–73.85%) highlight Nepal's diverse epidemiological environment. During the review period (2004–2022), Nepal's national deworming program did not experience interruptions; instead, it expanded to full national coverage and was later integrated into the Vitamin A “Plus” campaign to ensure biannual administration of albendazole [48]. Reported coverage has remained consistently high (commonly >90%, and >80% in recent estimates) [49], although minor variation persists in remote areas. However, these operational differences alone are unlikely to account for the spatial heterogeneity observed in our findings. Albendazole (400 mg single dose for children ≥2 years; 200 mg for ages 1–2 years) remains the primary drug used in mass drug administration, but its efficacy varies markedly by helminth species‐achieving very high cure rates for *A. lumbricoides* (>99%), moderate effectiveness against hookworm (≈82% to 96%), and substantially lower efficacy for *T. trichiura* (<50%) [50]. Importantly, albendazole has no therapeutic effect on protozoan parasites, which helps explain the persistence of protozoal infections even in areas with long‐standing deworming activities. These species‐specific pharmacologic differences, in combination with disparities in sanitation, water quality, and diagnostic sensitivity, provide a more plausible explanation for the spatial and temporal variation seen in our study than changes in MDA implementation. Despite these variations, the general downward trend continues to be a reliable gauge of the success of national initiatives to manage parasitic diseases.

The infection rates for both males and females decreased over time, with similar prevalence rates over all time periods. The prevalence decreased among males from 41.8% (2004–2010) to 24.7% (2010–2016) and then increased slightly to 27.9% (2016–2022). Similarly, the prevalence among females decreased from 40.4% to 24.6% and then slightly increased to 31.1% in the last period. Minimal sex‐related differences in the prevalence of GIPs have also been documented in previous studies conducted in Nepal and nearby regions [3, 9, 19]. This resemblance could be explained by similar hygiene habits between boys and girls in rural and urban areas, shared environmental exposures, and transmission through schools. On the other hand, a few isolated studies from western Nepal reported that males had somewhat higher infection rates [9, 19, 37, 51]. This could be because males spend more time outside and come into contact with soil, which increases their exposure to helminth eggs. On the other hand, research from eastern Nepal has occasionally reported a higher frequency among women [3, 20, 52], which could be related to household responsibilities that involve handling food and water. The lack of significant sex‐based variations in pooled estimates (adjusted prevalence: 12.7% for both sexes) indicates that behavioral and environmental factors, rather than biological sex, dictate exposure risk. This research highlights the necessity of community‐wide, as opposed to gender specific, programs that emphasize hygiene education, sanitation, and routine deworming for all school‐age children.

Different temporal patterns for helminth and protozoan infections were found by the meta‐analysis. HIs declined significantly from only 23.8% to 17.4% during the same period, but protozoan infections exhibited a substantial decline from 14.8% (2004–2010) to 7.4% (2016–2022). Protozoans showed a substantial decreasing trend (*β* = −0.077, *p* < 0.001), whereas helminths showed a minor increasing trend (*β* = 0.074, *p* < 0.001), according to logistic regression. Protozoan infections have decreased more sharply in tandem with advances in drinking water quality and hygiene awareness, both of which directly lower the level of fecal‐oral transmission of protozoa, such as *E. histolytica* and *G. lamblia* [9, 20]. The ongoing prevalence of helminths (average prevalence of 20.0%) is in line with earlier studies conducted in Nepal [7, 18, 25, 26, 27, 52] and other developing countries, such as India [53, 54, 55, 56, 57, 58], Bangladesh [59], and Pakistan [8], where poor footwear and inadequate sanitation lead to the persistence of GIPs such as *A. lumbricoides, T. trichiura*, and *Ancylostoma duodenale* [8, 16, 19, 28, 30, 60]. The varied patterns of helminth and protozoan infections indicate that interventions linked to water have been more successful than those related to soil. Reinfection, environmental resistance of helminth eggs, and insufficient MDA coverage can all lead to sustained helminth transmission. Consequently, integrated strategies‐combining deworming with sanitation enhancements, hygiene education, and the proper disposal of human waste‐should be prioritized in future control initiatives.

Several included studies revealed coinfections of helminths and protozoa, especially in rural areas [2, 8, 21, 25, 9, 29, 39], even though the current analysis focused mainly on overall and species‐specific prevalence. Multiparasitism frequently denotes inadequate access to healthcare and poor sanitation, which increases child morbidity [4, 17, 30, 60]. Coinfection rates can reach approximately 10–15%, according to studies conducted in mid‐ and far‐western Nepal [20, 26, 52, 61]. *A. lumbricoides* frequently co‐occurs with *G. lamblia* or *E. histolytica* [4, 5, 6, 28, 62]. Comparable trends have been noted in Ethiopia [5, 12, 42, 43, 63], where behavioral and environmental factors encourage polyparasitism [12, 17, 53]. Nonetheless, recent school‐based surveys conducted in Kathmandu and Pokhara revealed very low coinfection rates (<2%) [14, 25, 64, 65, 9], which is in line with urbanization, better cleanliness, and easier access to potable water. The fact that multiparasitism still exists in some rural areas highlights how unevenly public health advancements have been distributed throughout Nepal. Additionally, it emphasizes the value of integrated surveillance systems that can track several parasite species at once as opposed to concentrating on controlling a single infection.

In urban areas, the pooled prevalence was 29.0% (95% CI: 19.2%–41.4%), which was slightly higher than the 27.9% (95% CI: 19.8%–37.8%) reported in rural areas. With large confidence intervals indicating significant variation, the prevalence rates were 29.0% in urban areas and 27.9% in rural areas. This unexpectedly higher burden in urban settings may reflect factors, such as population overcrowding, inadequate sewage infrastructure, or increased risk of cross‐contamination, despite generally better access to healthcare and safe drinking water. Additionally, the greater number of studies conducted in urban areas could have contributed to this elevated estimate. Our analysis revealed a differential distribution of parasite types across urban and rural settings. Urban areas presented a greater proportion of protozoan parasites, whereas HIs were more common in rural populations. This observation aligns with findings from previous studies that reported elevated helminth prevalence in rural regions compared with their urban counterparts, although the evidence remains inconsistent across all contexts. In Nepal, fecal contamination of water sources is frequently reported in both rural and urban communities, including in municipal centers. In many urban areas, residents rely on shallow groundwater for household use due to unreliable piped water systems, potentially exposing them to fecal pathogens, such as *Giardia*, *Cryptosporidium*, and *E. histolytica*. These environmental vulnerabilities may help explain the persistence of protozoan infections even in urban settings where basic services are comparatively better.

As revealed by previous reports, the narrow differences in the prevalence of GIP infection in urban and rural areas could be associated with Nepal's growing urbanization without proper consideration of improvements in sanitation infrastructure [11, 17, 18, 27, 52, 66]. Recent reviews on some of the neglected food‐ and water‐borne parasites (*Cyclospora* and *Cryptosporidium*) in Nepal have highlighted their prevalence in humans of various age groups, including children, in relation to environmental exposure and other transmission variables [67, 68]. The conditions in peri‐urban settlements and slum regions are often comparable to those in rural areas. Studies from industrialized countries such as India [53, 54], on the other hand, demonstrate a clear urban‐rural split, with a significantly lower prevalence in metropolitan areas. The absence of such a split in Nepal indicates that public health has not yet improved proportionately with urban growth. The necessity for context‐specific interventions that target urban informal settlements, where issues with waste management, overcrowding, and inadequate drainage persist, as well as rural sanitation, is highlighted by the convergence of infection levels in these two areas.

Our review (2004–2022) offers a more thorough and current analysis that captures the impact of recent national health interventions, improved sanitation, deworming programs, and diagnostic advancements enacted after 2016, even though Kunwar's study on GIPs among Nepalese school children covered data only up to 2015 [24]. Our study investigated long‐term epidemiological trends, identified new patterns and regional variances, and reassessed the efficacy of control efforts by extending the timeframe to 2022. As a result, our research significantly contributes to the literature by providing a current understanding of the diminishing tendencies of GIPs and assisting with future policy and intervention initiatives in Nepal. Although direct epidemiological evidence from Nepal is limited, climate‐related factors are likely to influence GIP transmission patterns. A recent study in bovines from the Annapurna region reported higher gastrointestinal parasite prevalence at warmer, lower elevation zones [69], and global modeling studies similarly predict that rising temperatures and changing rainfall patterns will expand transmission windows for STHs [70]. These findings suggest that climate change may increasingly shape the geographic and seasonal distribution of GIPs in Nepal.

Several limitations should be acknowledged when our findings are interpreted. Although we estimated relative changes in infection patterns across consecutive 6‐year periods, these estimates are intended to reflect temporal trends rather than predict future outcomes beyond the study window. Furthermore, owing to inconsistencies in how studies reported protozoan and helminth prevalence separately, we calculated their relative proportions on the basis of the total number of parasites identified. Although this measure offers insight into the comparative burden of each parasite type, it does not represent true prevalence rates. The underreporting of certain parasites, particularly coccidian protozoa, such as *Cyclospora* and *Cryptosporidium*, and helminths, such as *E. vermicularis* and *S. stercoralis*, is likely because most of the studies did not employ the robust laboratory techniques necessary for their detection. Additionally, the reliance on the less sensitive direct wet mount method in some surveys may have further underestimated the prevalence of infection. Moreover, the geographic distribution of the studies was uneven, with a concentration in Nepal's central and western regions and limited representation from the midwestern and far‐western areas, which are often characterized by poorer sanitation infrastructure and limited access to toilet facilities. In addition, almost none of the included studies were conducted in the Himalayan highlands, preventing meaningful comparison of GIP prevalence across Nepal's three ecological zones (Himalayan, midhill, and Terai) and underscoring the need for future surveys that more fully represent high‐altitude regions. This imbalance underscores the need for more geographically inclusive research to ensure comprehensive national estimates. Furthermore, several of the included studies relied solely on direct wet‐mount or basic concentration techniques, which have limited sensitivity. These methods are unable to reliably detect a number of important parasites, including coccidian protozoa, such as *Cryptosporidium* and *Cyclospora*, or low‐intensity infections of helminths, such as *S. stercoralis*. As a result, the true prevalence of certain infections is likely underestimated, and reported species distributions should be interpreted with this diagnostic constraint in mind. Consequently, the observed decline in overall GIPs may not reflect true temporal patterns for helminths and protozoa that are poorly captured by standard diagnostic methods, and trends for these pathogens should be interpreted with caution.

## 5 Conclusion

In summary, our findings indicate a notable decline in the prevalence of GIPs among school‐aged children in Nepal over the past 18 years. Between 2004 and 2010, the pooled prevalence was 43.4%, which decreased to 28.3% during the 2016–2022 period. The burden of protozoan infections was more prominent in urban settings, whereas HIs were more common in rural areas. Additionally, polyparasitism, or coinfection with multiple parasites in children, has been reported in several instances, although at lower proportions. This overall reduction in GIPs likely reflects the positive impact of sustained public health measures aimed at improving sanitation, hygiene practices, and child health across the country.

## Author Contributions

**Jitendra Gautam**: conceptualization, formal analysis, visualization, investigation, methodology, software, writing – original draft. **Niten Bharati**: formal analysis, visualization, software, writing – original draft. **Shristi Bhandari**: methodology, writing – review and editing. **Darwin Niroula**: methodology, formal analysis, writing – review and editing. **Anisha K.C**.: review and editing. **Pitambar Dhakal**: writing – review and editing. **Kishor Pandey**: writing – review and editing. **Oskar Nowak**: writing – review and editing. **Rajendra Prasad Parajuli**: formal analysis, software, visualization, review and editing.

## Funding

The authors have nothing to report.

## Ethics Statement

The authors have nothing to report.

## Conflicts of Interest

The authors declare no conflicts of interest.

## Data Availability

Data supporting the findings of this study are available from the corresponding author upon reasonable request.
